# Using Analytic Hierarchy Process to Assess Beekeeping Suitability in Portuguese Controlled Areas: A First Approach

**DOI:** 10.3390/insects15020091

**Published:** 2024-01-29

**Authors:** Natália Roque, Paulo Fernandez, Carlos Silveira, Miguel Vilas-Boas, Ofélia Anjos

**Affiliations:** 1 IPCB—Agrarian School, Polytechnic Institute of Castelo Branco, 6000-084 Castelo Branco, Portugal; nroque@ipcb.pt; 2 CERNAS-IPCB—Research Centre for Natural Resources, Environment and Society, Polytechnic Institute of Castelo Branco, 6000-084 Castelo Branco, Portugal; 3 MED&CHANGE—Mediterranean Institute for Agriculture, Environment and Development & CHANGE—Global Change and Sustainability Institute, Universidade de Évora, 7006-554 Évora, Portugal; 4 Centro de Investigação de Montanha (CIMO), Instituto Politécnico de Bragança, Campus de Santa Apolónia, 5300-253 Bragança, Portugal; carlos.silveira@ipb.pt (C.S.); mvboas@ipb.pt (M.V.-B.); 5 Laboratório Associado para a Sustentabilidade e Tecnologia em Regiões de Montanha (SusTEC), Instituto Politécnico de Bragança, Campus de Santa Apolónia, 5300-253 Bragança, Portugal; 6 Centro de Estudos Florestais (CEF), Laboratório Associado TERRA, Instituto Superior de Agronomia, Universidade de Lisboa, 1349-017 Lisboa, Portugal

**Keywords:** beekeeping, suitability maps, GIS, multi-criteria analysis, fuzzy AHP, spatial planning

## Abstract

**Simple Summary:**

Beekeeping is an activity that supports agriculture and wildlife on earth, so it is very important to provide accurate information that is able to help in increasing the profitability of beekeeping and valorize pollination services. In this paper, a new methodology is proposed to assess beekeeping potential using Geographic Information Systems (GIS) and the Analytical Hierarchy Process. The proposed tool will support decision making by selecting the best apiary locations to maximize honey production and, consequently, the risks of losing bee colonies will be reduced. The Suitability map (apiary aptitude map) allows for validating the best locations for apiaries (considering the spatial and floristic resources use), and the aptitude for installing new apiaries or moving existing ones. In this sense, the application of Multi-criteria GIS Analysis, in the context of beekeeping planning, is a useful tool for the Beekeepers Association to manage honeybee resources within the territory in order to maximize their profit, and for government agencies to implement promotional measures and policies to maximize food support needs and mitigate spared diseases.

**Abstract:**

Beekeeping management is greatly influenced by spatial factors (e.g., land use/land cover, roads, or electrical energy networks), so GIS are a powerful tool to overlap and relate a variety of spatial data levels and, consequently, a very useful tool for beekeeping activity planning. This study was developed within the intervention area of three controlled zones managed by Portuguese Beekeepers Associations. The methodology, based on multi-criteria decision analysis, integrates several criteria, such as hydrographic networks, road networks, soil occupation, solar radiation, and electromagnetic radiation sources. These criteria were proposed and evaluated through online questionnaires carried out with beekeepers. Concerning the selected criteria and the respective geographical data, the most relevant were land use/land cover and water availability, with a significance of 44% and 24%, respectively. The beekeeping suitability map enabled us to evaluate the degree of compliance for the actual location of apiaries, with 60% of the apiaries being installed in high potential areas. In the context of beekeeping planning, the potential of the techniques applied seems to be an important tool for optimizing the location of apiaries and the profitability of beekeeping.

## 1. Introduction

Nowadays, beekeeping is widely recognized as an important environmental and socioeconomic activity linked to the sustainable development of rural areas. From an economic point of view, it offers an additional income to beekeepers through the provision of goods, such as honey, pollen, propolis, and royal jelly, but economic benefits for the local community are also expected since the pollination services provided by honeybees play a key role in the increased yield of agricultural crops [1,2,3,4]. Bees pollinate approximately 90% of flowering plants worldwide. Of the primary food crop varieties, 75% rely on pollinators for their growth [5]. For European crops, 84% of production for consumption is significantly dependent on pollination services [5,6]. Among the pollinators, the honeybee (*Apis mellifera* L.) is considered the most significant insect, given its potential for honey production and crop pollination [7,8]. Some authors have reported that 33% of pollination is carried out by honeybees [9,10,11]. From an environmental perspective, ecosystem services go beyond pollination, since pollinators ensure the conservation of wild plant species. In turn, these plant–insect interactions are also dependent on the air temperature, the number of plant species with available nectar or pollen, and their flowering period. In addition, honeybees are able to regulate the microclimate of their colonies within the internal temperature range of 33–36 °C to favour brood development [12,13]. In contrast, when there is a small abundance of melliferous plants and a short flowering period, the land suitability for beekeeping and consequent agricultural production is limited [13,14].

Nevertheless, despite the innumerable advantages associated with beekeeping activity, its subsistence is being seriously threatened due to anthropic and biotic stresses. Climate change, the fragmentation of habitats resulting from land use changes, the occurrence of diseases and insect pests, bee health, and side effects of the use of pesticides are some negative aspects that can contribute to significant losses in pollinating insect colonies worldwide, thus compromising the beekeeping and agricultural production, which are the main income sources for many farmers who live and work in rural areas [2,3,14,15,16,17,18,19,20,21]. To halt and reverse this trend, in 2018, the European Commission adopted the first-ever European Union (EU) framework on wild pollinators, called the EU Pollinators Initiative. This initiative, revised in January 2023, set long-term strategic objectives and actions by 2030 to be implemented at different levels and involving stakeholder groups to protect pollinators and their habitats [22]. Among the actions outlined to boost the beekeeping sector, the connections with other economic activities, namely ecotourism and its expansion to urban areas, have been investigated. Pantoja (2017) [23] designed a decision support system to link beekeeping and tourism, thus establishing priority areas for shared activities, whereas Stange et al. [2] developed a mapping and assessment approach to define urban honeybee zoning using a biophysical capacity model of the urban landscape to support pollinating insects.

To assess beekeeping suitability, Geographic Information Systems (GIS) are a useful geospatial technology to overlap, relate, process, and analyze a variety of spatial data layers, aiming to achieve mapping by suitability classes. GIS data models considering environmental and socioeconomic aspects (e.g., weather conditions, orography, land use/land cover, and distances from settlements, roads, and water) for beekeeping planning have been designed in several research studies [14,24,25,26,27,28]. In line with the GIS, a set of decision making support techniques, based on multi-criteria decision analysis (MCDA), has been developed and applied to find the best solutions within multiple alternatives.

AHP can be used for several purposes, such as for application in the selection of plant types on the community agroforestry land [29], for the selection of agricultural irrigation systems [30], and for the prioritization of absent quarantine pests [31], among many other applications. For beekeeping analysis, the most used MCDA techniques are the Analytical Hierarchy Process (AHP), the Technique of Order of Preference by Similarity to Ideal Solution (TOPSIS), the Preference Ranking Organization Method for Enrichment of Evaluations (PROMETHEE), and the fuzzy logic approach. Integrating MCDA into GIS enables the development of a GIS-based multi-criteria decision support framework for evaluating beekeeping suitability. Within the MCDA techniques, AHP is the most commonly used for spatial decision support in the identification of suitable locations for potential apiaries. AHP is an expert-driven modeling approach that considers the insights of beekeepers and other industry professionals to classify different suitability criteria. The resulting weights derived from these inputs are then used to generate beekeeping suitability maps. To validate these maps, various factors are considered, including the existing locations of apiaries, the number of beekeepers, honey production, and the analysis of honey samples [8,23,32,33,34,35,36,37].

In the interest of capturing landscape dynamics, remotely sensed features have been retrieved to determine suitable apiary locations considering the effects of seasonality. Otto et al. [16] developed habitat selection models that cross land-cover and land-use changes with their influence on the apiary site selection. In another research study [37], the Normalized Difference Vegetation Index (NDVI), derived from satellite images, was combined with a crop coverage registration system to establish migratory apiaries according to flowering seasons, and thus benefit from pollen and nectar source plants that contribute to increased honey yields. Under future scenarios, the land suitability for beekeeping has also been evaluated, and a considerable expansion of urban areas and a consequent decrease in the beekeeping potential areas are estimated [17]. Beyond land use changes, higher negative impacts for beekeeping in future will be expected, mainly due to thermal stress [38].

Given this state-of-the-art framework for beekeeping suitability, which shows relevant scientific advances and concerns within the sector, certain issues that are addressed in the present study still require further investigation:

(i) First of all, it is necessary to extend the number of studies to EU member countries, thus corroborating with the Pollinators Initiative;
(ii) When determining priority areas (i.e., with the highest suitability classes) not only should the preservation of pollinating insect densities be considered, but also the satisfaction of crop pollination needs;
(iii) In addition to identifying priority areas for apiary locations, land suitability maps should provide information on prohibited areas for beekeeping in accordance with legal constraints;
(iv) Finally, for greater accuracy in the resulting suitability maps, a broad knowledge of the target regions is essential to identify the criteria to be used and their relative importance.

Therefore, the main objective of this research is to assess the beekeeping potential of Portuguese controlled areas using GIS-AHP techniques, in order to support decision making in the selection of the best locations for apiaries, thus maximizing honey production and reducing the risk of bee colony losses. From another perspective, since the study areas have a large agricultural expression, an increase in pollination and subsequent crop yield will be expected. It is important to highlight that this approach could easily be replicated in other territories, as long as the criteria and their relevance to beekeeping suitability are identified.

## 2. Materials and Methods

### 2.1. Study Areas

**Figure 1:**
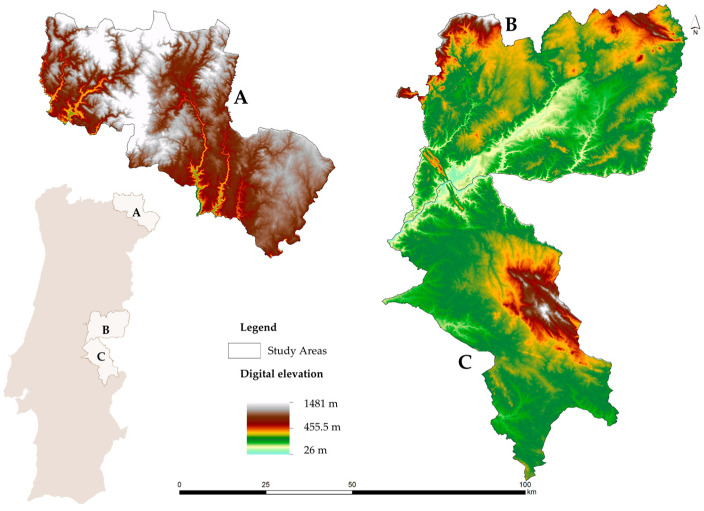
Geographic location of the study areas and their management entities: (**A**) Beekeepers Association of the Natural Park of Montesinho (AAPNM); (**B**) Beekeepers Association of the International Tejo Natural Park (Meltagus); (**C**) Beekeepers Association in northeast of Alentejo (Apilegre).

To assess the beekeeping potential, three Portuguese controlled areas, where there is a systematic control of bee diseases and high diversity of bee flora, were selected (Figure 1). These controlled areas comply with the EU regulations, Directive 92/65/EEC [39], and with the recommendations from the World Organization for Animal Health, which define animal health policies and regulate the trade and importation of derived products. In turn, the high biodiversity of these areas, especially of melliferous plant species, can be explained by the fact that they are integrated into protected areas, thus receiving special protection due to their recognized natural, ecological, and cultural values. In total, the study areas cover 860,000 ha and are managed by three beekeepers associations.

Area A is located in northeastern Portugal, covering the municipalities of Bragança, Vinhais, Vimioso, and Miranda do Douro (284,000 ha), which are managed by the Beekeepers Association of the Natural Park of Montesinho (AAPNM). The relief of this beekeeping territory is very rugged, with elevations in the dense hydrographic network, where the Azibo dam (250 m) stands out, to mountainous areas, such as Montesinho (1486 m), Nogueira (1319 m), and Coroa (1274 m). From a climatic point of view, considering the 30-year average climatological normal (1971–2000) for the meteorological station of Bragança (41°48′ N, 6°44′ W, 690 m altitude) [40], the average annual temperature was 12.3 °C, varying monthly from 4.4 °C in January to 21.3 °C in July. In the coldest month, average extreme values were between 0.3 and 8.5 °C, whereas in July, these temperature extremes ranged from 14 to 28.5 °C. In terms of precipitation, the annual average was 758.3 mm, distributed seasonally from 18.4 mm in August to 118.6 mm in December. This area contains, among others, the climatophilous Eurasian oak woods (*Quercus robur* subsp. *broteroana, Fagaceae*) and the Mediterranean oak woods (*Q*. *pyrenaica*). There are also cork oak formations (*Q. suber*). There are also two subspecies of carqueja (*Pterospartum tridentatum*), a low shrub that is undetectable in mountain heathland. To the east, the boundary runs parallel to the valley of the Douro International River, along the border between the climatophilous holm oak forests (*Q. rotundifolia*) characteristic of the Salmantine Sector and the Lusitanian–Durian forests of cork oak or black oak. The characteristic landscape is made up of a mosaic of oak woodland, gypsum woodland, heath or heath-steppe, chestnut groves, and apple orchards. Potatoes, rye, and barbel wheat were also very popular in the past. Also noteworthy are the evergreen *Quercus* woods, the cistus, the *genisteae*, and Mediterranean perennial crops such as vines, olive groves, almond trees, and, more recently, pistachios [41].

Area B is located in the center inland of Portugal, more specifically in the municipalities of Castelo Branco, Idanha-a-Nova, and Vila Velha de Rodão (318,000 ha), and is managed by the Beekeepers Association of the International Tejo Natural Park (Meltagus). In its orography, the Gardunha (1220 m) and Monsanto and Penha Garcia (750 m) mountains stand out. Regarding the climate, data recorded from the meteorological station of Castelo Branco (39°50′ N, 7°28′ W, 386 m altitude) for the period 1971–2000 show annual averages of temperature and precipitation around 15.7 °C and 758.3 mm, respectively. On average, the minimum temperature for the coldest month (January) was 3.9 °C, whereas the maximum for the hottest month (July) was set at 32.1 °C [40]. This area is dominated by climatophilous forests of cork oak (*Quercus suber*), black oak (*Q. pyrenaica*), and, residually, holm oak (*Q. rotundifolia*). There are also stretches of mesomediterranean oak woodland from Beira Baixa. These riparian forests are less frequent on the Aravil, Erges, and Ponsul rivers, where the ash trees (dominated by *Fraxinus angustifolia*) and *Salix salviifolia* willows are much more widespread. Agroforestry in terms of the tree layer, which is more or less sparse, is made up of the species of the Quercus genus mentioned above and resulted from an alteration of the primitive forests that probably began in the Palaeolithic period. Its herbaceous layer is made up of pastures of the *Poetea bulbosae*, *Tuberarietea guttatae* or Stipo giganteae-Agrostietea castellanae classes, but depending on grazing management, its floristic diversity may be impoverished and it may become semi-initrophilous pastures of *Stellarietea mediae* [41].

Area C is a contiguous territory to area B, which includes the municipalities of Portalegre, Nisa, Crato, Castelo de Vide, Marvão, Monforte, and Arronches (258,000 ha), under the management of the Beekeepers Association in the northeast of Alentejo (Apilegre). The São Mamede mountain (1021 m) and the Tejo river (40 m), which also crosses the beekeeping area B, represent the extremes of terrain elevation. Taking as reference the meteorological station of Portalegre (39°17′ N, 7°25′ W, 597 m altitude) and the same historical period (1971–2000) [40], average annual values of 15.2 °C and 852.4 mm for the temperature and precipitation variables were recorded. Concerning the temperature range, the minimum value for the coldest month (January) was 5.7 °C, whereas the maximum temperature in the hottest month (July) reached 29.8 °C. This area corresponds mainly to cork oak forests, *Quercus suber* and *Quercus rotundifolia*; sometimes to deciduous (marcescent) oak forests: *Quercus faginea* and *Quercus marianica*; and, more rarely, on temporarily hygrophilous soils, these oak forests may be accompanied by *Quercus robur* ssp. *estremadurensis*. The cork oaks or oak forests can also include other trees, such as the *Quercus rivasmartinezii*, the carob tree, Ceratonia siliqua or the *Olea europaea* ssp. Other trees, such as *Crataegus monogyna*, *Pyrus bourgaeana* or *Juniperus oxycedrus* ssp., and *J. turbinata*, can also occur [41].

Regarding the rainfall regime, the desired condition is not observed from June to September in the three study areas, given the summer aridity associated with the strong Mediterranean influence [40]. In addition to climate dependence, beekeeping is also significantly influenced by the existing bee flora, since its diversity and availability contribute to providing the needed quantities of nectar and pollen, enabling the proper development of bee colonies and the regular collection of beekeeping supplies [42,43,44]. Table 1 presents the main plant species that occur in the study areas [41]. These plants have a high economic value for beekeeping and ensure a relatively constant income throughout the year due to their distinct flowering phenological records.

### 2.2. GIS Multi-Criteria Decision Analysis Approach

The criteria are a set of guidelines or requirements used as the basis for a decision and are classified as factors or constraints. A factor is a criterion that enhances or detracts from the suitability of a specific alternative for the activity under analysis. A constraint serves to limit or restrict the alternatives under consideration; therefore, it is an element or feature that represents limitations or restrictions and whether an area is considered unsuitable.

The set of criteria used for beekeeping suitability analysis was based on theoretical principles indicated in the literature, experts’ opinions, and legal constraints. According to Wolff et al. [46], the ideal apiary site should be away from urban, industrial or commercial areas, near fresh water supplies but away from swamping or flooding valleys, near food sources, and with accessible rural roads. Taking these considerations into account, the following baseline criteria were categorized into two groups:

(i) Factors—orography (elevation, slope, and aspect), land use/land cover (agriculture, forestry, and shrubs), meteorological conditions (solar radiation, air temperature, and precipitation), road and hydrographic networks, proximity to water, and apiary locations [46];
(ii) Constraints—proximity to urban settlements and road networks, electromagnetic radiation sources, and density of apiaries [20,21,47,48].

Factors consider environmental and socioeconomic variables that drive the beekeeping activity, whereas constraints represent limitations to the activity arising from the current legislation. In the Portuguese legislative framework, the Decree Law n.° 203/2005 of 25 November [48] establishes that apiaries must be located more than 50 m from public roads and 100 m from any building in use. Rural and agricultural roads, as well as buildings used to support beekeeping activities, are excluded from this legal impediment.

Regarding the data sources, the following spatial data sets were used in the study:

- Orographic characteristics, which are related to flora and meteorological conditions, were derived from the Digital Terrain Model (DTM) with a 25 m spatial resolution [49];
- Land use/land cover data were extracted from the vector land cover/use map of Mainland Portugal for 2018 (COS2018), whose minimum mapping unit is 1 ha [50]; Table S1 shows the classes used from the national classification;
- Meteorological variables were retrieved from Monteiro-Henriques et al. [51];
- Road and hydrographic networks were derived from the Open Data Portal [52,53,54];
- Electromagnetic radiation sources, in relation to electric power transmission and distribution towers, were provided by the power distributor on its open data platform [55];
- The solar radiation mapping over the terrain was based on the DTM, following the radiation model developed by Hofierka and Súri (2002) [56];
- Apiary locations were established using a portable Global Navigation Satellite System (GNSS) receiver, Trimble JUNO 5 Series, with sub-metric accuracy (Trimble, Sunnyvale, CA, USA). This geospatial survey with GNSS receiver also served to collect information on the existing flora near the apiaries.

Considering these factors, beekeeping suitability tends to improve as follows:

- Apiaries should be located in rural areas with a high diversity of bee flora, thus ensuring the well-being and health of bees and the consequent increase in honey production;
- For their survival and productivity, bees must have a clean water source, so it is considered beneficial that beehives are located less than 500 m from this natural resource [42];
- Beehives located in areas with solar radiation between 500 and 1200 W.m^−2^ show an increase in bee activity, which is more pronounced on slopes with greater and more prolonged sun exposure [12];
- In terms of accessibility, the proximity to rural roads is considered the most advantageous option for the implantation of apiaries, while safeguarding the legal distances [48];
- Finally, to avoid possible issues with the beekeeper’s activity, the effect of electromagnetic radiation sources was taken into consideration. Some research suggests a possible effect on the orientation abilities of the bees, as well as other animals and humans [47,57]. Therefore, in our study, it was defined that beekeeping activity should be established with a 250 m radius from the medium- and high-voltage power networks, and a 500 m radius from the very high voltage power networks and mobile communication antennas [58].

### 2.3. Data Analysis

Once the baseline criteria are identified, we intended to assess how these can be integrated into the study areas and ascertain their relative importance in determining beekeeping potential. Thus, taking advantage of the knowledge of the sector’s professionals, particularly beekeepers who develop their activity in the target territories, the following specific criteria were considered:

1. Availability of bee flora;
2. History of bee diseases;
3. Solar radiation;
4. Slope orientation;
5. Wildfire risk index;
6. Proximity to rural roads;
7. Proximity to water surfaces;
8. Distance to ionizing radiation sources (e.g., mobile communication antennas);
9. Proximity to landfills.

#### 2.3.1. Experts’ Opinions

**Figure 2:**
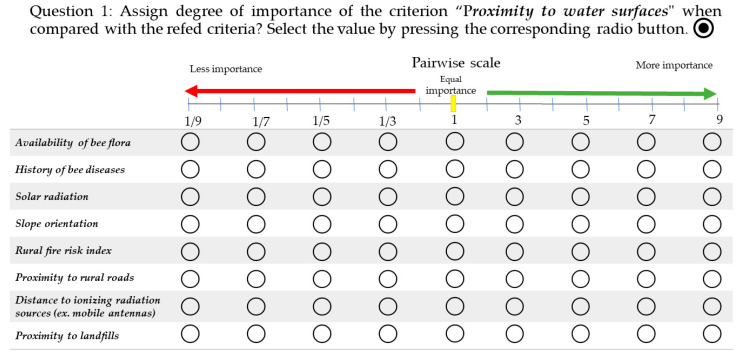
Pairwise comparison scale for the AHP preferences matrix. An example of the questions included in the online survey directed to the beekeepers.

In addition to the selection of specific criteria, beekeepers played a key role in weighing their relative importance for beekeeping suitability mapping. To that end, an online survey directed to beekeepers was carried out. The survey consisted of two parts: (i) in the first one, a personal characterization was requested, and a brief introductory note on the evaluation and weighting criteria according to the AHP pairwise scale (Figure 2) was presented; (ii) the second part was composed of nine closed questions that allowed for a comparison between criteria. Throughout the questionnaire, the AHP pairwise comparisons were made by asking the experts: “Which of the two factors contributes more to beekeeping suitability?”. The response options were taken according to a nine-point numerical scale developed by Saaty (2008) [59], which defines the importance of each criterion on a numerical scale from 1 to 9, where values below 1 mean less importance than the target criterion, 1 is defined as of equal importance, and higher values indicate that the analyzed criterion is more important than the target one (Figure 2). An example of the type of question is shown in Figure 2.

In total, 27 surveys were fully completed, most of which by men in the 35–44 age group. Regarding apiary size, 41.6% of the respondents carry out the activity for self-consumption (lower than 25 hives), followed by 29.2% who are professional beekeepers (higher than 75 hives). Among the respondents, 16.7% do not have an apiary, but are dedicated to research in this field of activity.

After treating and analyzing the survey’s results, a panel of experts, composed of members of the national beekeeping federation, the regional directorate of agriculture, and university experts, met to discuss the final criteria to be considered for beekeeping potential mapping. Accordingly, based on the nine criteria previously identified by the experts and which formed part of the survey, criteria 2, 4, 5, and 9 were excluded due to their correlation with other criteria or the fact that the data scale was not standardized.

#### 2.3.2. Analytic Hierarchy Process AHP-GIS Data Model

**Figure 3:**
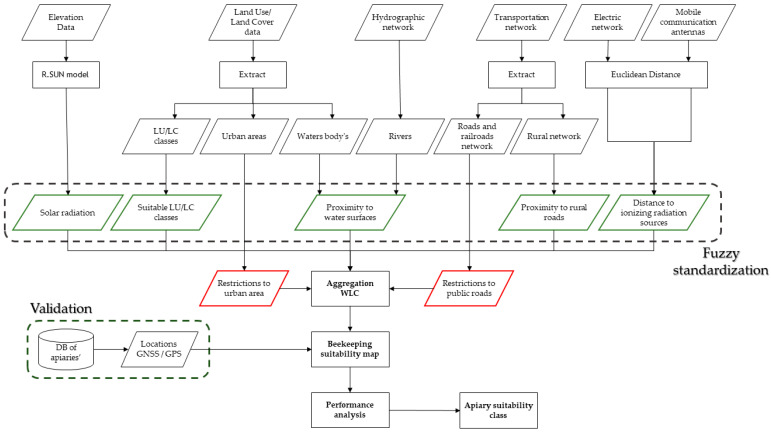
Flowchart of the GIS data model combined with AHP multicriteria analysis. Criteria are marked in green and restrictions in red.

The AHP-GIS model shown in Figure 3 presents the process and tools implemented in GIS, adapting the methodology of Saaty (2008) [59].

The flowchart shows the tools and their applications in transforming data sources into criteria, applying the AHP-GIS model followed by fuzzy normalization, which precedes WLC aggregation. Finally, to obtain the apicultural suitability, the restricted areas were extracted in accordance with the legal framework.

The assessment process aims to determine the location of apiaries that do not comply with the legal framework or whether it is possible to relocate them and maximise their suitability to benefit from relocation to nearby areas. The validation process considers the location of the apiaries promoted by the beekeepers’ association.

**Figure 4:**
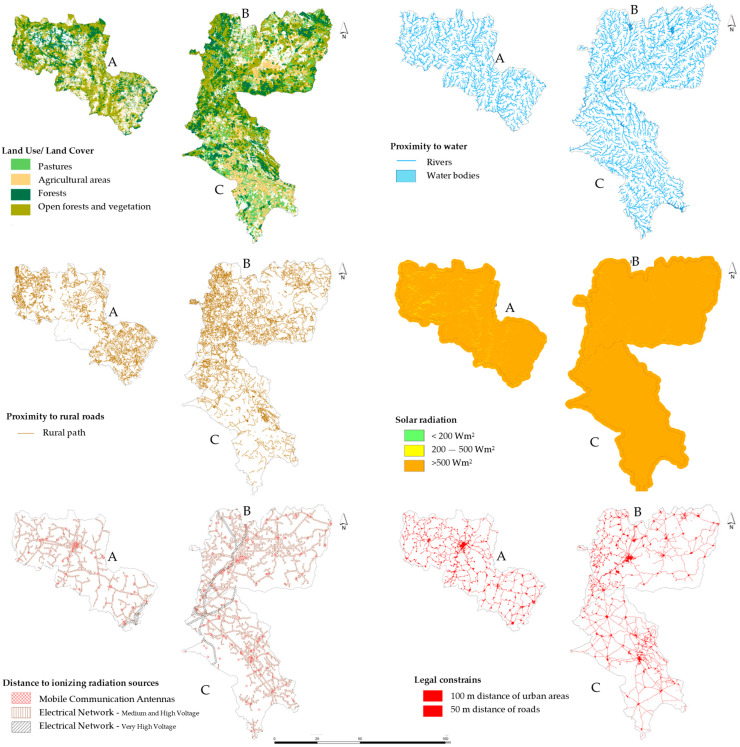
Spatial representation of the criteria used to assess the beekeeping potential in the controlled study areas: (**A**) Beekeepers Association of the Natural Park of Montesinho (AAPNM); (**B**) Beekeepers Association of the International Tejo Natural Park (Meltagus); (**C**) Beekeepers Association in northeast of Alentejo (Apilegre).

As a result of Section 2.3.1 Expert opinions, a set of criteria that summarize the factors and constraints of greatest importance for beekeeping activity were defined. These criteria were used in the AHP-GIS data model and are represented spatially in Figure 4, and Table S1 (Supplementary Materials) shows the suitable Land Use and Land Cover (LULC) classes and respective descriptions proposed in this classification.

This method determines the preferences between the sets of criteria, one pair at a time, making comparisons between pairs using the Saaty scale. Once these comparisons have been made for all the pairs of criteria, the AHP matrix is constructed, the matrix is normalized, and the vector of priorities is determined. It is through this pairwise comparison that the AHP method is usually obtained, as it reduces the complexity of the problem. This method has two internal validations, the Consistency Index (CI) and the Consistency Ratio (CR). Equation (1) is used to calculate CI, the consistency coefficient of the pairwise comparison matrix:

(1) $$\mathrm{CI}=\frac{\lambda_{\max}-n}{n-1}$$

where λ_max_ is the largest eigenvalue of the matrix, and n is the size of the comparison matrix AHP in the analysis.

After calculating the CI, it is possible to obtain the CR, consistency ratio, using Equation (2):

(2) $$\mathrm{CR}=\frac{\mathrm{CI}}{\mathrm{RI}}$$

where the random index (RI) is a tabulated value that varies with the number of criteria (n); based on expert knowledge when the CR exceeds 0.1, it is necessary to carry out a revision of the pairwise comparison matrix with different values [59].

#### 2.3.3. Beekeeping Suitability

To assess the beekeeping suitability of the targeted territories, the methodological procedure was based on the following steps: (i) the hierarchical structure of the beekeeping suitability; (ii) the standardization of the criteria; (iii) criteria weighting; and (iv) the aggregation and mapping of beekeeping potential.

(i) Hierarchical structure of the beekeeping potential model

Once the final criteria were defined, they were compared with each other based on the AHP preferences matrix (Figure 2). This assessment, from the selection of the criteria to their hierarchization, was carried out by a panel of beekeeping experts. Table 2 shows the results of this comparison, highlighting that the most relevant criteria are land cover, proximity to water, and solar radiation and that the least relevant criterion is distance from electromagnetic radiation.

(ii) Standardization of the criteria

**Figure 5:**
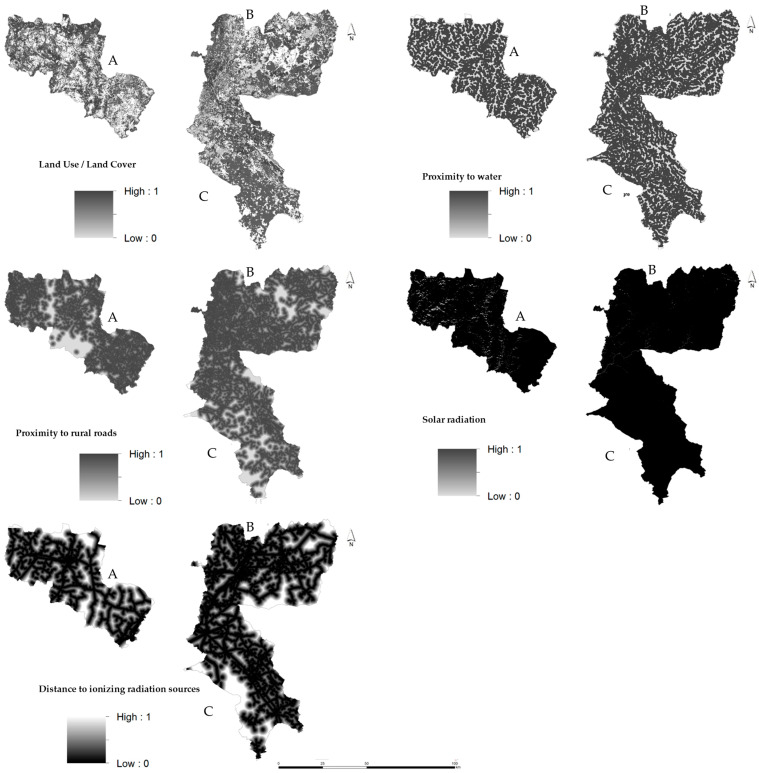
Spatial representation of the fuzzy standardization: (**A**) Beekeepers Association of the Natural Park of Montesinho (AAPNM); (**B**) Beekeepers Association of the International Tejo Natural Park (Meltagus); (**C**) Beekeepers Association in northeast of Alentejo (Apilegre).

Since the integration and comparison of criteria are conditioned by the fact that they have different scales of measurement, it is necessary to normalize them on a common scale. Since the criteria are qualitatively and quantitatively measurable, it is necessary to carry out fuzzy standardization and transform the criteria into a continuous numerical scale, where the values vary between 0 and 1. The fuzzy approach proposed by Boroushaki and Malczewski [60] was applied to convert the spatial data/criteria to be included in the analysis, as shown in Figure 5.

(iii) Criteria weighting

The purpose of assigning weights to the criteria is to express the degree of importance of each factor in relation to the other ones and this constitutes a challenging step in the evaluation and decision-making process. The relative weight of the vulnerability criterion was estimated by the weighted linear combination (WLC) method, originally developed by Saaty [59].

The WLC is completed when the CR value is less than 0.1. In this model, a CR of 0.077 was obtained by Equation (2), with the vector of eigenvalues λ_Max_ = 5.346 and IC calculated by Equation (1) of 0.086, concluding the process of this matrix, referred to in Section 2.3.2 Analytic Hierarchy Process (GIS-AHP) data model.

The AHP compares pairwise criteria and then computes the overall relative weights based on aggregate calculations of all pairwise ratios [61].

(iv) Aggregation and mapping

Finally, the criteria were aggregated using the weighting of the criteria on the normalized maps, in which each of the maps had a weight, as presented in Table 3. This aggregation process is also known as an additive combination of simple weighting, which multiplies the scores of the criteria for each alternative [62]. The restricted area was subtracted from this result, which constitutes the map of beekeeping potential on a continuous scale between 0 and 1.

## 3. Results and Discussion

**Figure 6:**
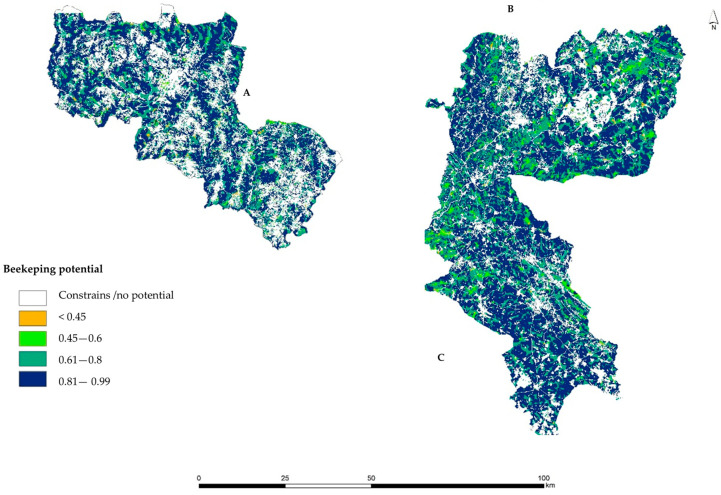
Beekeeping suitability maps for the controlled study areas: (**A**) Beekeepers Association of the Natural Park of Montesinho (AAPNM); (**B**) Beekeepers Association of the International Tejo Natural Park (Meltagus); (**C**) Beekeepers Association in northeast of Alentejo (Apilegre).

The information on AHP weightiness, through the pairwise comparison matrix crossed with the WLC method (Table 2 and Table 3), was used to produce beekeeping suitability maps for the controlled study areas (Figure 6).

As mentioned before, suitability was translated in a normalized scale between 0 and 1, where higher values represent ideal locations for the installation of apiaries. Looking at Figure 6, it can be seen that there are many areas without beekeeping potential (i.e., a value equal to 0), which correspond to the areas where at least one of the criteria is absent or null. When beekeeping suitability is recognized in the target territory, its spatial distribution varies between 0.26 and 0.99. Lower suitability values are associated with values below 0.45, whereas higher suitability was determined in locations with over 0.80. In addition to beekeeping suitability, areas unsuitable for the practice of this activity were also mapped in Figure 6, considering the legal constraints. In this study, restricted areas (i.e., constraints) for beekeeping correspond to the perimeter based on the safety distances defined in the national legislation (Decree-Law n.° 203/2005), namely 100 m around the urban settlements and 50 m away from public roads.

To facilitate the interpretation of the suitability results and support decision making, five classes were established that relate the standardized numerical values to the qualitative assessments (Table 4). As a first analysis, it should be emphasized that 72% of the studied territory has beekeeping suitability potential, thus justifying the commitment to control these areas for the development of beekeeping activity.

The model using AHP techniques provides more accurate results when we substrate the legal restricted areas to the potential suitability areas, and the results show that high suitability areas (High and Very high classes) sum up to 68.4% of the total area. The class *Very high* covers 46% of the area and class *High* covers 22.3%; for the remaining classes, *Medium* (≅3%) and *Low* (≅1%), the scores are in accordance with the classes’ percentages of the total area.

Moreover, in each class, it should be noted that the suitability area in relation to the corresponding total results from the elimination of sites that do not comply with the current legislation, defined as restricted areas.

The No potential class represents 23.6% and occupies 202,508 ha. It is the third largest class as a percentage of the total area. In terms of restrictive areas, it represents the largest class 3.3%, which accounts for 45.5% of all restrictive areas, essentially due to its spatial proximity to restrictive conditions.

**Figure 7:**
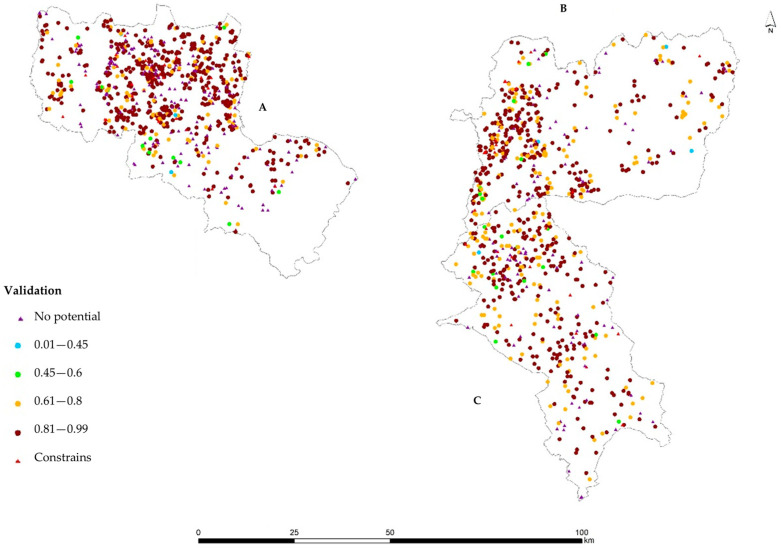
Estimated beekeeping suitability for the sites where the apiaries are located. Controlled study areas: (**A**) Beekeepers Association of the Natural Park of Montesinho (AAPNM); (**B**) Beekeepers Association of the International Tejo Natural Park (Meltagus); (**C**) Beekeepers Association in northeast of Alentejo (Apilegre).

When crossing the beekeeping suitability map with the location of existing apiaries, to check whether the apiaries were located in higher beekeeping potential areas (Figure 7), it was concluded that the majority of the apiaries were installed in areas classified with very high suitability (56.6%), representing 1101 of the total 1946 apiaries.

The High class represents 17.2% and situated 335 apiaries, while the Medium and Low classes were residual with 0.8% and 1.5%, corresponding to 14 and 29 apiaries, respectively. The No Potential class accounts for 18.3% of apiaries, corresponding to 358 apiaries.

Non-compliance classes represent 5.6%, which is 109 apiaries, in areas legally prohibited for beekeeping activity, with 66 apiaries from zone A (Figure 7), 28 in the zone B area, and 15 apiaries in zone C. The availability of these results for the management entities of the controlled areas will allow a more targeted follow-up for civil parishes with greater insight of diseases or where the quality factors of the honey can be improved.

Therefore, a suitability assessment can be extremely helpful in planning land uses, merging a wide range of unrelated information to produce datasets where areas are ranked by their suitability for a certain activity, according to specific requirements [14,19]. In addition, determining suitable locations for beekeeping should be evaluated in the field of land use planning considering economic, ecological, environmental, and social aspects within a spatiotemporal perspective [26,32,35].

Beekeeping suitability maps can also be used by government agencies to implement various promotional measures and policies, as well as by beekeepers to identify suitable sites for apiary locations and relocations in order to maximize their profit.

Zoccali et al. [14] performed beekeeping suitability maps in Calabria, Italy, based on aptitude in rank classes and used fuzzy logic to normalize the variables used, similarly to the methods used in this study. However, this study does not validate the data with the location of the apiaries and does not take into account the opinion of the beekeepers and experts for the AHP methodology.

Other important study made in Konya, Turkey, performed the suitability map of beekeeping also based on aptitude classes in ranks, but did not use fuzzy logic to normalize the variables used. The advantage of this study is the validation of the maps with the honey production and density of colonies which improve their interest. Additionally, the aforementioned authors do not incorporate the beekeepers and experts, which is an advantage of the present study [35].

The AHP is also used to create a map of forage flora and its flowering period and it compares different methods of creating suitability maps using remote sensing; the methods were compared using validation grids with more than 600 points but, for the assessment of potential, just over 20 points were considered for the validation of the class with the greatest potential [36].

In a very interesting study by Sari [17], they created beekeeping potential maps as a function of the LULC for predicting suitability for beekeeping in future scenarios. With this model, it was possible to infer the efficiency in terms of modeling land use and land cover and to predict future solutions for the same area. The data corroborate the model that we tested.

Another study similar to the present study was performed in Adiyaman, Turkey [32], whereby the authors created a suitability beekeeping map with only four classes, but the most relevant data show that a quarter of the study area has no beekeeping suitability and the classes with the highest suitability represent 58% of the territory; our data follow a very similar line, whereby 68% of the area has beekeeping potential and nearly a fifth of the area has no suitability.

Notwithstanding the achievements reported here on beekeeping potential mapping, there are still methodological aspects to be refined. The next steps involve (i) a more detailed characterization of the existing floral diversity; (ii) the restructuring of the questionnaire based on a wider participation of beekeeping stakeholders; (iii) the testing of other criteria that may influence beekeeping activity; (iv) improving the spatial resolution of these criteria; (v) replicating the adopted multi-criteria decision analysis approach in other beekeeping territories; and (vi) collecting information about the current honey production associated with the sites where the beekeeping potential was determined.

The methodology used in this paper exhibits characteristics and approaches that have proven effective in a specific region of Portugal. However, we believe that this approach could also be successfully replicated in other regions of Portugal, or even in different countries, thereby optimizing the results and effectiveness of the process in diverse contexts.

## 4. Conclusions

This research focused on assessing suitability for beekeeping in three Portuguese controlled areas. It employed Geographic Information Systems (GIS) and Analytical Hierarchy Process (AHP) techniques to create a decision support framework for selecting optimal locations for apiaries. The goal was to enhance honey production and minimize bee colony losses. A noteworthy aspect of this study was the collaboration between beekeepers associations and beekeeping experts, who contributed to the selection and weighting of criteria, thereby improving the reliability of the resulting beekeeping suitability maps. Initially, a set of criteria was established based on the literature and experts’ input, and this was refined through online questionnaires and meetings with local beekeepers to create a methodological structure for the target territories.

The study assessed various criteria, with the highest importance given to Land use/land cover (44%) and Proximity to rivers and water bodies (24%). The key findings indicate that 68.4% of the studied territory has a high or very high suitability for beekeeping, where most existing apiaries are located. For apiaries in areas with low (5.6%) or medium suitability, relocating them is recommended to enhance profitability.

Regarding legal constraints, most beekeepers adhere to regulations regarding apiary placement, including safety distances around urban areas and roads. Mapping prohibited beekeeping areas helps prevent conflicts and supports sustainable rural development.

From a methodological perspective, the integrated GIS-AHP approach, coupled with support from beekeepers associations, can be effectively applied in other regions. This approach aids policymakers, planners, beekeepers associations, and beekeepers in promoting underexploited beekeeping areas, preserving sustainable practices, and adapting to climate change scenarios.

## Figures and Tables

**Table 1:** List of the most abundant plants in each study area [45].

Area A	Area B	Area C
*Quercus pyrenaica*	*Scirpoides holoschoenus*	*Quercus suber*
*Andryala integrifolia*	*Cyperus longus*	*Cistus ladanifer* subsp.* ladanifer*
*Lavandula pedunculata* subsp.* pedunculata*	*Briza maxima*	*Quercus pyrenaica*
*Quercus rotundifolia*	*Cistus ladanifer* subsp.* ladanifer*	*Pinus pinaster*
*Convolvulus arvensis*	*Quercus rotundifolia*	*Arbutus unedo*
*Fraxinus angustifolia* subsp.* angustifolia*	*Oenanthe crocata*	*Urginea maritima*
*Crataegus monogyna*	*Bromus hordeaceus*	*Cistus salviifolius*
*Cistus ladanifer* subsp.* ladanifer*	*Mentha suaveolens*	*Narcissus bulbocodium subsp. bulbocodium*
*Rubus ulmifolius* var.* ulmifolius*	*Lavandula pedunculata* subsp.* pedunculata*	*Ruscus aculeatus*
*Chondrilla juncea*	*Agrostis castellana*	*Alnus glutinosa*
*Daphne gnidium*	*Pinus pinaster*	*Andryala integrifolia*
*Sedum forsterianum*	*Rumex induratus*	*Aristolochia paucinervis*
*Cytisus multiflorus*	*Chamaemelum mixtum*	*Briza maxima*
*Olea europaea* var.* europaea*	*Dittrichia viscosa* subsp.* viscosa*	*Pteridium aquilinum* subsp.* aquilinum*
*Ornithopus compressus*	*Plantago lanceolata*	*Trifolium stellatum*
*Trifolium campestre*	*Iris xiphium* var.* lusitanica*	*Anarrhinum bellidifolium*
*Hypochaeris radicata*	*Quercus suber*	*Arenaria montana* subsp.* montana*
*Achillea millefolium*	*Mentha pulegium*	*Carduus tenuiflorus*
*Trifolium glomeratum*	*Brachypodium phoenicoides*	*Cistus crispus*
*Hypericum perforatum*	*Hymenocarpos lotoides*	*Cistus psilosepalus*
*Trifolium pratense* subsp.* pratense*	*Ornithopus compressus*	*Crataegus monogyna*
*Briza maxima*	*Silene gallica*	*Digitalis thapsi*
*Sanguisorba verrucosa*	*Andryala integrifolia*	*Geranium molle*
*Clinopodium vulgare*	*Adenocarpus lainzii*	*Narcissus triandrus*
*Pteridium aquilinum* subsp.* aquilinum*	*Tuberaria guttata*	*Origanum vulgare* subsp.* virens*
*Petrorhagia nanteuilii*	*Anarrhinum bellidifolium*	*Quercus rotundifolia*
*Jasione montana*	*Trifolium angustifolium*	*Rubus ulmifolius* var.* ulmifolius*
*Leontodon taraxacoides* subsp.* longirostris*	*Fraxinus angustifolia* subsp.* angustifolia*	*Cephalanthera longifólia*
*Rumex acetosella* subsp.* Angiocarpos*	*Lythrum salicaria*	*Ceterach officinarum* subsp.* officinarum*
*Trifolium angustifolium*	*Echium plantagineum*	*Ferula communis* subsp.* catalaunica*

**Table 2:** Results of the pairwise comparison matrix.

Criteria	Land Use/Land Cover	Proximity to Water	Solar Radiation	Proximity to Rural Roads	Distance to Ionizing Radiation Sources
Land use/Land cover	1	3	3	5	7
Proximity to water	1/3	1	1	5	7
Solar radiation	1/3	1	1	7	7
Proximity to rural roads	1/5	1/5	1/7	1	3
Distance to ionizing radiation sources	1/7	1/7	1/7	1/3	1

**Table 3:** Relative weights applying the weighted linear combination (WLC) method.

Criteria	Weight (%)
Land use/Land cover	43.5
Proximity to rivers and water bodies	24.1
Solar radiation	21.9
Proximity to rural roads	6.8
Distance to electromagnetic radiation sources	3.7

**Table 4:** Total and functional (suitability) areas by beekeeping suitability class and restricted areas (%) considering the geographic coverage of the study areas.

Beekeeping Suitability Classes	Total Area		Potential Suitability		Restricted Areas	
	(ha)	(%)	(ha)	(%)	(ha)	(%)
No potential [0–0.26]	202,508	23.6	173,851	20.3	28,657	3.3
Low [0.26–0.45]	9191	1.1	8616	1.0	575	0.1
Medium [0.45–0.65]	28,193	3.3	26,040	3.2	2153	0.1
High [0.65–0.80]	204,266	23.8	191,738	22.3	12,528	1.5
Very high [0.80–1]	415,553	48.2	397,048	46.0	18,505	2.2
Total	859,713	100	797,287	92.7	62,418	7.3

## Data Availability

Data are contained within the article and supplementary materials.

## Supplementary Materials

The following supporting information can be downloaded at: https://www.mdpi.com/article/10.3390/insects15020091/s1. Table S1: Suitable land use and land cover (LULC) classes and respective description.
