# Supplementary material for: Using Analytic Hierarchy Process to Assess Beekeeping Suitability in Portuguese Controlled Areas: A First Approach
Source: Insects. 2024 Jan 29;15(2):91. doi: 10.3390/insects15020091 (PMC10888816; doi:10.3390/insects15020091)
Supplement: Supplementary file 1 [file insects-15-00091-s001.zip › insects-2697141-supplementary.pdf]

*Article*

# Using Analytic Hierarchy Process to assess beekeeping suitability in Portuguese controlled areas: A First Approach

**Natália Roque[1], Paulo Fernandez[1,2,3*], Carlos Silveira[4,5], Miguel Vilas-Boas[4,5] and Ofélia Anjos[1,2,6*]**

1 IPCB, Polytechnique Institute of Castelo Branco, 6000-084 Castelo Branco, Portugal; nroque@ipcb.pt (N.R.); palex@ipcb.pt (P.F.); ofelia@ipcb.pt (O.A.)

2 CERNAS-IPCB Research Centre for Natural Resources, Environment and Society, Polytechnic Institute of Castelo Branco, 6000-084 Castelo Branco, Portugal

3 MED&CHANGE— Mediterranean Institute for Agriculture, Environment and Development & CHANGE – Global Change and Sustainability Institute, Universidade de Évora, 7006-554 Évora, Portugal

4 Centro de Investigação de Montanha (CIMO), Instituto Politécnico de Bragança, Campus de Santa Apolónia, 5300-253 Bragança, Portugal; carlos.silveira@ipb.pt (C.S.); mvboas@ipb.pt (M.V.B.)

5 Laboratório Associado para a Sustentabilidade e Tecnologia em Regiões de Montanha (SusTEC), Instituto Politécnico de Bragança, Campus de Santa Apolónia, 5300-253 Bragança, Portugal

6 Centro de Estudos Florestais (CEF), Laboratório Associado TERRA, Instituto Superior de Agronomia, Universidade de Lisboa, 349-017 Lisboa, Portugal

* Correspondence: Ofélia Anjos: ofelia@ipcb.pt and Paulo Fernandez: palex@ipcb.pt

**Table S1**: Suitable land use and land cover (LULC) classes and respective description.

| LULC classes | Description |
|---|---|
| Grassland | Areas with or without human intervention occupied by vegetation, essentially of the herbaceous type, whether cultivated (sown) or natural (spontaneous), that are not included in a rotational farming system and occupy an area equal to or greater than 25% of the surface. |
| Heterogeneous agricultural areas | Agricultural areas with various types of associations between temporary crops, pastures, permanent crops, and areas of forest and/or natural vegetation and/or wetlands and/or bodies of water. It includes temporary crops and/or pastures associated with permanent crops, temporary or permanent crops cultivated under forest cover, areas of mosaics of temporary crops, pastures, and permanent crops, and landscapes where crops and pastures are mixed with natural or semi-natural areas. |
| Agroforestry | Agroforestry surfaces consist of the association (vertical association in the same plot) of temporary crops and/or pastures (improved or poor spontaneous) and/or permanent crops with forest species with a canopy coverage equal to or greater than 10%. |
| Forestry | Lands with forestry use, occupied by forest trees, or temporarily cleared as a result of cultural cuts or extraordinary cuts due to biotic disturbances (pests, diseases) or abiotic disturbances (fires, storms). That includes: Cork oak, Other oaks, Chestnut, Eucalyptus, Deciduous forests and Pine woods. Trees originating from natural regeneration, seeding, or planting must reach a height greater than or equal to 5 meters and, as a whole, have a canopy coverage greater than or equal to 10%. The understory is not dedicated to agriculture or recreational activities when integrated into an urban context. This includes lands occupied by live or dead forest trees resulting from natural regeneration, seeding, or planting, clear-cutting, new forest plantations, and areas recently affected by forest fires in the process of regeneration for less than 5 years. |
| Shrublands | Natural areas of spontaneous vegetation, either sparse or dense, where shrub cover (e.g., heather, blackberries, broom, gorse, wild olive). It includes abandoned olive groves if there are fewer than 45 trees/ha. |
| Sparsely vegetated areas | Natural areas with little or no vegetation including recently burnt areas and bare rock. Cloud include shrub and herbaceous vegetation occupies if the [10% to 25%], and include areas where the surface covered by trees is less than 10%. |
